# Supplementary material for: Getting More Out of Biomedical Documents with GATE's Full Lifecycle Open Source Text Analytics
Source: PLoS Comput Biol. 2013 Feb 7;9(2):e1002854. doi: 10.1371/journal.pcbi.1002854 (PMC3567135; doi:10.1371/journal.pcbi.1002854)
Supplement: Dataset S1 — GATE software. Dataset S1 bundled with this paper contains a distribution of GATE (or see http://gate.ac.uk/download/). (TGZ) [file pcbi.1002854.s001.tgz › plos-gate/plugins/Gazetteer_LKB/doc/samples.html]

The Large KB gazetteer - Samples


---

Last Published: 2009-10-17

---

##### Large KB Gazetteer

- Overview
- Usage
- Q & A
- GATE Compatibility
- GATE Gazetteer Feature Comparison
- Semantic Enrichment PR
- **Samples**

##### Developer Documentation

- Dictionary Lifecycle

The large gazetteer KB comes with a few samples and the a companion PR that showcases the usefulness of using Linked Data URI as identifiers in the gazetteer. All samples are in the *samples* sub-directory of the plugin.

## Samples

- dictionary\_from\_local\_ontology - A sample of the primary use-case of the gazetteer - dictionary, loaded from local RDF files. In this sample, the RDF files contain a taxonomy. The query that loads all classes with their names is in *dictionary\_from\_local\_ontology/query.txt*.
- dictionary\_from\_remote\_ontology - A sample of loading a dictionary from a public Linked Data server - in this case the LDSR. Loads 10000 names of entertainers.
- sample\_pipelines
  - pipeline.gapp - Very small pipelines that initializes a gazetteer with the dictionary from *dictionary\_from\_local\_ontology*. Sample documents are included.
  - sample\_linked\_data\_mashup.gapp - Loads a gazetteer with the entertainers from *dictionary\_from\_remote\_ontology*, finds entertainers in the sample documents, and then runs the Semantic Enrichment PR. It adds the birthplace of every looked up entertainer.

---

©
2009
Ontotext AD

---
